# Memories: Failures and Dreams by Alain de Weck · a Book Review

**DOI:** 10.1097/WOX.0b013e3181a5d3a1

**Published:** 2009-05-15

**Authors:** Johannes Ring

**Affiliations:** 1 Munich, Germany

## 

Professor Alain de Weck, born 1928, has written his memoirs, reason enough to recommend the book for members of the World Allergy Organization: Alain de Weck was a member of the Board of Directors for almost 30 years, organized an unforgettable World Congress 1988 in Montreux, and was President of the organization from 1991 to 1994.

These memoirs are different from typical autobiographical notes where famous people tend to promote themselves and stress their importance.

Alain de Weck does quite differently. On the basis of his personal experience, he reflects on many things in life--much more than the history of allergy!

The basic steps toward the molecular recognition of allergic responses were taken not only in protein biochemistry but also in the discovery of Immunoglobulin E (IgE) antibodies. At the same time, the model of penicillin allergy contributed greatly to the understanding of anaphylaxis. It was Alain de Weck's work, when he started in the laboratory of Herman Eisen in St. Louis, that finally led to the concept of hapten inhibition as a preventive modality in allergy. In those days it became clear that monovalent binding does not induce allergic reactions, but bridging of antibodies on the surface of the mast cell has to occur. Later on, Alain de Weck built up his Institute for Allergology and Clinical Immunology at the University of Bern, where he was engaged in various projects such as developing better diagnostic techniques with *in vitro *cellular assays and sulfidoleukotriene release from basophils after priming with Interleukin 3, T cell regulation, and IgE and anti-IgE antibodies.

He describes how tuberculosis was treated in the 1930s before streptomycin and how doctors were living in Switzerland in the mountains. He also writes about the development of allergy and immunology in the second half of the 20th century, of all the pitfalls and great steps of progress. He vividly reflects on the culture in science, university life, and academic research. The reader receives in-depth information on the life of academic societies, not only the World Allergy Organization (WAO), formerly the International Association of Allergology and Clinical Immunology, but also the International Union of Immunologic Societies (IAACI), both of which Alain de Weck served as president--a rather rare coincidence in personal union! Other societies like the Collegium Internationale Allergologicum (CIA), the European Academy of Allergology and Clinical Immunology (EAACI), the Swiss Society, and the German Society are mentioned.

Furthermore, the reader learns much about Switzerland and life in Switzerland in the 20th century. Alain de Weck is a critical personality and this book has surely not been censured. He dares to mention names and illustrate facts even when they do not seem so favorable.

He gives his very subjective, but well-observed, impressions of many countries in the world where he has traveled or worked; he quite openly comments on both the good and the less attractive characteristics of the different nationalities.

He gives a classification of different typologies of researchers including the "recognition seeker," the "hair splitter", and the "traveling salesman".

Of course, readers accompany this great man through 80 years and learn a great deal, not just from his travel adventures in China, Mongolia, and Africa.

At the end, he dares to give "inner court" reflections on important matters like men and women, thoughts which are often missing in official autobiographies.

Taken together, this autobiographical book will bestow great pleasure to its readers--and not just to allergists and immunologists; but for them it should be a "must read."

Johannes Ring, MD, PhD

Munich, Germany

## Note

ISBN: 9783868052190, Pro Business, Berlin (2008).

