# Memories: Failures and Dreams by Alain de Weck. A Book Review: Erratum

**DOI:** 10.1097/WOX.0b013e3181b0e443

**Published:** 2009-06-15

**Authors:** Alain de Weck

## 

In the article "Memories: Failures and Dreams by Alain de Weck. A Book Review," which appeared in volume 2 of *The World Allergy Organization Journal *on page 62, the name Arthur Eisen is incorrect. This should appear as Herman Eisen.

This error has been noted in the original version of the article, which is available at http://www.waojournal.org.
